# Effect of introducing virtual community and community group buying on customer’s perceived value and loyalty behavior: A convenience store-based perspective

**DOI:** 10.3389/fpsyg.2022.989463

**Published:** 2022-09-26

**Authors:** Xiaoyu Xu, Zhineng Hu

**Affiliations:** Business School, Sichuan University, Chengdu, China

**Keywords:** LP design structures, virtual community experience, perceived value, customer loyalty, community group buying, social media marketing

## Abstract

Customers’ declining receptivity to conventional marketing tools has been a challenge for convenience stores. To overcome this, retailers are turning to social media as a new, potent marketing tool for creating business prospects and encouraging direct customer interaction. However, it is still unknown how social media marketing affects the shifts in customer behavior. This paper expands on the relationship of “loyalty program (LP) + virtual community experience → perceived value → customer loyalty” in the traditional convenience store scenario, refining the variables of virtual community experience, perceived value, and customer loyalty. It also compares the effectiveness of different LP design structures (reward amounts × reward time limits) and explores the mediation impact of program loyalty and the moderation effect of alternative attractiveness. The results demonstrate the superior performance of LPs with an expiry policy and differential returns and highlight the importance of enhancing members’ virtual community experiences in fostering customer perceived value and loyalty. The results also show the minor negative moderation impact of community group buying and prove that emotional value significantly impacts customer loyalty. Still, the social value does not affect program loyalty. The recommendations are offered, such as designing growing-oriented and periodical zeroing LPs, as well as using new social media marketing tools (virtual community-based marketing) to empower traditional marketing techniques (LP-based relationship marketing) and constructing a “convenience store + community group buying” model. The findings have substantial theoretical and practical implications for traditional convenience stores in properly implementing loyalty and social media marketing tactics to maximize customer value and loyalty with a limited budget.

## Introduction

Internet and social media are progressively becoming an indispensable part of modern life, and they have significantly altered retail business potential, purchase procedures, and customer shopping behaviors (Chen and Lin, 2019). According to the 49th Statistical Report on Internet Development in China [China Internet Network Information Center (CNNIC), 2022], there were 1.032 billion Internet users and 1.007 billion social media users in December 2021, with 842 million online shoppers accounting for 81.6% of all Internet users. The online retail sales reached 13.1 trillion CNY, accounting for 29.7% of the total retail sales of consumer goods. Many traditional retail stores have realized the importance of constantly optimizing existing marketing tools and introducing social media marketing in response to an increasingly competitive environment and the rise of online consumption (Pantano and Dennis, 2019).

Retailers have relied on LPs as one of the most widely recognized marketing techniques to reward customers in long-term relationships and drive repeat purchases by delivering short-and long-term rewards. The quantity, size, and breadth of LPs have increased dramatically, and LP study has piqued the interest of many academics. Numerous academic studies have examined the appeal of LPs and the positive influence of programs on customer behavior (Bazargan et al., 2017; Bruneau et al., 2018; Shaikh et al., 2018). There has been little study on the usefulness of various program structures. More research is needed to determine which designs impact customers’ perceptions of value and motivation to adopt and use the LP. Furthermore, it is unclear which type of experience and values customers perceive when using LPs will improve the program’s success and increase customer loyalty to the store. Yet, it is a significant problem for retailers.

Introducing social media marketing tools, such as virtual communities, presents more significant opportunities and challenges for traditional brick-and-mortar stores. Stores are disconnected from customers after they leave the store in an era without mobile Internet, so many stores rely on introducing LPs to build long-term customer relationships. With the development of mobile technology and the change in customer demand, only relying on traditional marketing tools cannot meet the needs of the new era of customer groups. Nowadays, many stores have established virtual communities relying on social media platforms (e.g., WeChat, QQ, Tik Tok, etc.), thus keeping in touch with customers anytime and anywhere. Compared with the traditional LPs, the store can publish product information and transfer the store’s core value in various forms such as text, pictures, voice, and video in the communities, so that customers can know more about the product and the store without going to the store (Huangfu et al., 2022). Customers’ multi-directional information in the communities can also enhance communication and give them more authentic and reliable feelings (Qiao et al., 2019).

Previous studies have proved that the establishment of virtual communities can improve the value perception of customers and then enhance the purchase intention of customers, but there is little research on the specific mechanism of this phenomenon (Kim W. et al., 2018; Huangfu et al., 2022). Moreover, not all communities are thriving. To attract a large number of customers, most virtual communities do not set the entry threshold, resulting in mixed community members and poor internal interaction and sharing. Releasing too much promotion or advertising information in the community will cause members’ aversion, and irregular promotional activities in the community can only increase customer activity in a short time (Lu et al., 2021). However, LPs can help remedy these shortcomings by screening loyal customers with high value and making targeted marketing plans accordingly.

The rise of the “home economy” due to the COVID-19 quarantine policy and the changes in spending habits has accelerated the community economy’s development. Residents’ reliance on community commerce has grown due to its convenience, high frequency, and rapid adaptation to changes. Their daily consumption is gradually sinking to the “last mile” with residents turning to community convenience stores, supermarkets, and some new social e-commerce models (e.g., community group buying) developed based on social media technologies. The conventional offline community retail formats and new retail formats have influenced the traditional convenience stores’ market share. The growth rate of traditional convenience stores’ market share has decreased in recent years. Still, the downward trend is the least compared to other physical retail formats (mini-supermarkets, hypermarkets, department stores, etc.; iiMedia Research, 2022). In 2021, the market size of the convenience store industry reached 298.8 billion CNY, and its number reached 157,000. The concentration (one store for about every 9,000 people) is still low due to the large land area and uneven economic development between regions in China when compared with that of the United States and Japan (one store for about every 2,000 people; iiMedia Research, 2022). Therefore, convenience stores have ample development space. The research based on the influencing factors of customer loyalty under the convenience store scenario also has excellent theoretical and practical significance.

As a new scene derived from social e-commerce, community group buying has received special attention from retailers and scholars. Community group buying is a new shopping and consuming mode that connects customers to all areas of local life through a “collection + pre-sale + online booking + offline self-pickup” model, using acquaintances and social relationships as a bond (Li et al., 2022). It has overgrown because its convenient business model meets customers’ purchasing needs for cost-effective goods paired with social interaction. There were about 200 community, group-buying-related market players in China by the end of 2021 (e.g., Meituan Selected, TaoCaiCai, Xingsheng Selected, etc.), with market size of 120.51 billion CNY and 646 million users [Net Economic and Social E-commerce Research Center (WJS), 2022]. Academics have paid extensive attention to the emergence of community group buying, and current research focuses primarily on the qualitative study of the model’s benefits and drawbacks (Wang, 2017; Huang et al., 2021). However, convenience stores must evaluate the impact of these rising alternatives if they are to remain unbeatable in the increasingly fierce market battle.

This paper makes four contributions to customer loyalty research and social media marketing practice. First, we prove that LPs with different structures significantly differ in customer value perception. Namely, LPs with differential returns and an expiry policy substantially affect members’ perceived value more. Second, we confirm that the interaction between new social marketing tools (virtual community) and traditional marketing tools (LPs) significantly enhances customer perceived value and loyalty. Third, we illuminate the driving effect of different dimensions of perceived value on customer loyalty formation and discover that the loyalty to the program can transform into a more enduring form of loyalty to the store. Fourth, we also find that the emergence of new social e-commerce models such as community group buying impacts the relationship between customer perceived value and loyalty, but the impact is weak. These findings may help retailers to develop competitive LPs for success and fill a research void in the quantitative analysis on the impact of LP structure and social media marketing, the combination of different marketing methods, and the emergence of new social e-commerce.

The remainder of the paper is organized as follows. Section Literature review and hypotheses reviews the relevant literature and develops hypotheses that capture how LP structure, virtual community experience, and attractiveness of new retail formats impact members’ value perception and loyalty behavior. We then conduct a 2 × 2 quasi-experimental design and introduce the questionnaire design and the measurement of items in section Methodology and data collection. Section Statistical analysis and results shows the empirical research analysis process and results. The last section highlights the theoretical and managerial implications, discusses our research’s limitations, and identifies future research directions.

## Literature review and hypotheses

### Definition and research progress of members’ virtual community experience

With the rapid advancement of social media technology and the widespread usage of the Internet, online virtual brand communities are becoming increasingly popular among retailers due to their benefits of being time and space free as well as low cost. To better understand the genuine feelings of members in virtual communities, this section delves into the concept and the dividing dimensions of members’ virtual community experience. Although there is no single definition of virtual community experience, they all highlight customers’ whole perceptions and feelings along the process. Some research describes the virtual community experience as a succession of customer sensations comprising cognitive, emotional, behavioral, sensory, and social experiences before, during, and after purchase (Qiao et al., 2019; Huangfu et al., 2022). Some define virtual community experience as a thorough feeling and evaluation of each element’s cognitive, emotional, physical, sensory, and social experiences when customers experience a company’s products or services (Mclean et al., 2018). Different researchers have offered different division dimensions based on their study backgrounds; however, the most common dimensions include emotional experience, social experience, entertainment experience, hedonic experience, and interactive experience (Foroudi et al., 2016; Mclean et al., 2018; Huangfu et al., 2022).

This paper defines members’ virtual community experience as a collection of emotions and a comprehensive assessment of their interaction with stores or other members, the value of services, access to information, and the environment in the virtual community formed by stores. We then divided community experience into three dimensions, including information acquisition experience, recreational enjoyment experience, and social interaction experience. Information acquisition experience is the emotion generated by members’ access to knowledge about their needs and information sharing among them *via* the virtual community. When members of the community encounter problems while choosing products or using products, they can seek information or assistance from stores and other members, which can help them make decisions and solve problems more effectively. Recreational enjoyment experience is the emotional feeling of pleasure, relaxation, and excitement generated through browsing virtual community content and participating in community interactions and activities. Social interaction experience reflects members’ experience of friendship, affection, and other forms of social support gained through participating in community interaction. Members actively form close bonds, interact with one another at the level of extensive information or interpersonal relationships, and share their consumption experiences, product use experiences, and life stories. These experience strengthens their favorable feelings and preference for virtual communities and enhances their identification with brand values and stores.

### Definition and research progress of perceived value

#### Research progress on the definition and dimensions of perceived value

Perceived value significantly influences customers’ purchase decisions, social status perception, and the relationship between customers and stores (Wang et al., 2015). The research on perceived value drivers is significant for improving marketing activities’ effectiveness and maintaining stores’ competitive advantage. To create a successful LP, you must first understand how customers perceive the value and how that affects customer loyalty (Xie et al., 2015). The previous research defines perceived value as customers’ overall assessment and preference of the utility of the product and service and the perceived net benefit after considering the gains and costs (Zeithaml, 1988; El-Adly and Eid, 2016). In this paper, perceived value refers to members’ comprehensive evaluation of all the relevant benefits, rewards, and the time, money, and energy the membership spends.

Scholars continually study the dimensional structure of perceived value while providing a conceptual definition. Many previous studies have taken a one-dimensional approach and only measured economic benefit, failing to recognize its complex and multifaceted nature. However, financial benefits alone do not sustain long-term customer relationships, and customers can easily switch to competing offerings if competitors offer better financial benefits (Wu et al., 2014; Brashear-Alejandro et al., 2016). Recently, several researchers have used a multidimensional approach to conceptualize perceived value (Table 1), but there is no single classification standard.

**Table 1:** A literature review of perceived value dimensions.

Dimensions	Scholars	Dimensions	Scholars
Hedonic value and functional value	Li and Lee (2016)	Utilitarian value and hedonic value	Chiu et al. (2014)
Functional value, emotional value, and social value	Chen (2018)	Social value, hedonic value, and epistemic value	Yang and Lin (2014)
Functional value, psychological value, and external value	Koo et al. (2020)	Utilitarian value, hedonic value, and symbolic value	Dorotic et al. (2012)
Utilitarian value, hedonic value, and social value	Kim and Park (2013)	Economic value, functional value, emotional value, and symbolic value	Rintamäki and Kirves (2017)
Economic value, functional value, emotional value, and social value	Sweeney and Soutar (2001)	Quality value, value for money, emotional value, relational value, and customization value	Chuah et al. (2017)

This study divides perceived value into four categories based on the actual situation of convenience stores: economic value, functional value, social value, and emotional value. Functional value refers to the value obtained from the perceived quality or function of products or services, including product quality, product promotion activities, and member-exclusive service experience (Koo et al., 2020). Members can receive the most up-to-date product and service information on a regular and timely basis through virtual communities; they can also acquire qualify for discounts or rewards through LPs, decreasing their buy blindness and expense. Social value reflects the social status and special preferential treatment that members perceive compared with non-member customers when they enjoy exclusive membership activities and shopping discounts through participating in LPs (Bruneau et al., 2018). Social value also refers to the sense of belonging and social approbation recognition. Members can become partners with retailers, make friends, learn new things, and share consumption experiences and social resources through virtual communities, thereby expanding their social network (Kim and Park, 2013). Economic value emphasizes the benefits that the LPs provide to customers with a high cash value, which results mainly from a reduction in monetary sacrifice and the benefits from sales offers or discounted prices (Rintamäki and Kirves, 2017). Emotional value refers to intangible or psychic benefits, which can reflect enhanced mood, pleasure, enjoyment, and feelings of being comfortable (Chuah et al., 2017). The communication platform set up by LPs makes sharing possible, the interaction with the store increases members’ favor and recognition, and the communication with others fosters relationships and enjoyment (Rintamäki and Kirves, 2017).

#### Relationship between virtual community experience and perceived value

Many scholars have studied the relationship between members’ virtual community experience and perceived value. Experience marketing is essential for organizations to meet customers’ individualized needs and enhance their perceived value in a market context where product homogenization is increasing (Mclean et al., 2018). Joining a virtual community causes members to become part of an exclusive group of privileged customers, to identify with this group, and likely share associated values (Fullerton, 2014). A virtual community is an effective tool for modern businesses to cultivate high-value customers and promote their brands because there is a strong correlation between customers’ propensity to spend and their participation in virtual communities (Huangfu et al., 2022). Therefore, we predict that virtual community experience plays a vital role in forming customer value and advancing the following hypotheses.

*H1:* Members’ virtual community experience significantly and positively affects their perceived value.

Customers can access information resources specific to store members and acquire the information they need *via* conversation with others through virtual communities, regardless of geographical and time constraints. According to prior research, sharing and exchanging information in the community gives customers pleasure and satisfaction, raises their functional value, and meets their high-level needs (Qiao et al., 2019). Customers’ participation gives timely access to various product and service promotion information, effectively lowering the purchase cost (Chen, 2018). Customers’ access to product knowledge enriches and can simultaneously acquire functional, emotional, and social values through communicating and sharing information (Foroudi et al., 2016; Huangfu et al., 2022). Based on the above discussion, we propose the following hypotheses:

*H1a-d:* Members’ information acquisition experience in the virtual community significantly and positively affects their (a) functional value, (b) emotional value, (c) social value, and (d) economic value.

The virtual community’s social interaction experience gives customers a sense of belonging, importance, and integration, as well as meeting emotional demands (Mclean et al., 2018). More frequent communications allow members to enjoy the pleasures of communication and identity expression and provide customers with a specific social value (Lemon and Verhoef, 2016). The heterogeneity among members decreases as the amount of interaction and experience increases. Members will progressively transition from passively obtaining information to actively sharing it and invest more emotionally, cognitively, and behaviorally in the community. Taken together, we predict that members’ social interaction experience in the virtual community may positively affect the different dimensions of perceived value. Therefore, we propose the following hypothesis.

*H1e-g:* Members’ social interaction experience in the virtual community has a significant positive impact on their (e) functional value, (f) emotional value, and (g) social value.

Humans, as social animals, require social contact to belong to specific groups or organizations. Humans with the sociable human trait seek out organizational support and spiritual comfort. Keeping customers relying on product features in this customer-oriented era is becoming increasingly challenging. Improving the emotional value of customers is a more effective technique. Entertainment activities are needed for social humans to pursue happiness and relieve life pressure. Virtual communities’ intriguing interactions and many varied activities may promote joy and happiness or boost the feeling of enjoyment and meet members’ emotional requirements (Kim W. et al., 2018). Therefore, we propose the following hypothesis.

*H1h:* Members’ recreational enjoyment experience in the virtual community has a significant positive impact on their emotional value.

### Definition and research hypotheses of LP design structures

LP design structures are multifaceted and involve several elements, such as reward amount, type, timing, and redemption policies (Yang et al., 2019, 2021). There is no consensus on which type of reward amount is more effective and whether setting a time limit on reward redemption can bring actual loyal customers. Therefore, this paper mainly focuses on the effects of the reward time limits and reward amounts on customer loyalty cultivation in traditional convenience stores.

#### Definition and research progress of LP reward amounts

Reward amounts (refer to the reward that the LP delivers to members each time) available to members may influence their attitudes and participation enthusiasm; hence research into this topic is of great importance (Irina et al., 2022). The reward amounts are usually divided into equal returns and differential returns. Equal returns refer to a linear structure with no tiers and equal benefits for all members(Steinhoff and Palmatier, 2016). In contrast, differential return refers to a hierarchical structure with patterns of levels that members reach based on their consumption quantity. The return amounts offered vary based on their level (Zeng et al., 2022). Early research focuses on exploring the effectiveness of equal returns; however, more recent research has begun to challenge its usefulness and has shifted its focus to differential returns. Many empirical studies have shown that differential returns support better resource allocation and increase customer motivation to stay at the same level or fight for higher levels through increased purchases to achieve notable benefits (Steinhoff and Palmatier, 2016; Yang et al., 2021). Some also highlight its superiority in fostering feelings of status and arousing the desire for higher levels as members perceive their high status compared to lower levels, which contributes to a more excellent perception of higher levels (Eggert et al., 2015; Yang et al., 2020). These arguments suggest that LPs with differential returns motivate members’ perceived value more effectively. Therefore, this study proposes:

*H2:* Compared with the equal returns, LPs with differential returns have a more substantial positive effect on members’ perceived value.

*H2a-d:* Compared with the equal returns, LPs with differential returns have a stronger positive effect on the (a) functional value; (b) emotional value; (c) social value; and (d) economic value.

#### Definition and research progress of LP reward time limits

Whether to impose a time limit on reward redemption has sparked debate. Businesses frequently set reward expiration dates to avoid financial liabilities and decrease active involvement among members. At the same time, the fear of unpleasant customer experiences prompted some to extend reward expiration periods or implement a no-expiry policy (Bazargan et al., 2017). This paper investigates the influence of reward time limits (expiry vs. no-expiry policy) on customer loyalty.

According to previous studies, the duration of reward expiration time and the consequences of reward expiration strategy substantially impact LP performance. According to the target gradient theory, reward expiration has a beneficial impact on customer purchasing behavior, for it generates a time pressure mechanism by keeping customers engaged with LPs and leading to more purchases in the period leading up to the due date for award redemption (Dorotic et al., 2014). Customers are motivated to maintain or improve their relationship with retailers based on their gain/effort ratio. The perceived practical, hedonic, social, and functional benefits represent the retailer’s efforts (Mimouni-Chaabane and Volle, 2010). Reward expiry policies may encourage reward redemption within the time restriction and maximize customer perception of reward, which can help them establish loyalty and stay active, resulting in higher-value customers (Evanschitzky et al., 2015). In summary, we predict that LPs with an expiry policy are more effective in motivating members’ perceived value. Therefore, we hypothesize the following:

*H3:* Compared with the no-expiry policy, LPs with an expiry policy have a more substantial positive effect on members’ perceived value.

*H3a-d:* Compared with the no-expiry policy, LPs with an expiry policy have a more substantial positive effect on the (a) functional value, (b) emotional value, (c) social value, and (d) economic value.

### Definition and research progress of customer loyalty

The development of LPs has been motivated by the shift in marketing strategy toward a customer-centric emphasis. The introduction of LPs enhances sales revenue and establishes customer loyalty by encouraging repeat purchases, understanding customers’ purchasing preferences, and carrying out targeted marketing activities (Berezan et al., 2017). However, prior research has primarily focused on program loyalty, with only a few studies delving deeper into the different forms of loyalty (Kang et al., 2015; Tanford, 2016). This article distinguished program loyalty from shop loyalty to further examine the role of LPs and prevent overestimating their effects.

#### Definition and relationship between program loyalty and store loyalty

Program loyalty is economical and transactional and is fueled by program incentives (Gupta et al., 2018). Program loyal members who demand more product benefits and membership privileges are susceptible to retail price fluctuations (So et al., 2013). They are less inclined to pay a higher price for the stores’ products and are more likely to defect to a competitor’s program that offers more appealing benefits (Yang et al., 2019). Store loyalty is an emotionally motivated, relational form of loyalty that entails members’ more profound attachment and identification with the store and their behavioral desire to make repeat purchases, spread positive word-of-mouth, and pay a premium (Gupta et al., 2018). Store loyal members are more inclined to pay higher prices. They are less swayed by competitive offerings because they strongly desire to maintain a long-term relationship and a strong sense of loyalty to the store (Brashear-Alejandro et al., 2016).

Many previous studies have researched the relationship between program loyalty and store loyalty. Attachment to LPs has been proved to influence the affective nature of the customer-store relationship and positively affect store loyalty (Pandit and Vilches-Montero, 2016). Customers’ overall identification with the store has also increased as they become loyal to LPs, leading to better identification and prompting them to create closer ties with the brand (Konuk, 2018). Firms are recommended to foster program loyalty, which translates into a more profound and enduring sense of brand loyalty (Gupta et al., 2018). Therefore, we advance the following hypothesis:

*H4:* Program loyalty has a significant positive impact on store loyalty.

#### Relationship between perceived value and customer loyalty

In terms of member perceived value and its relationship to loyalty, numerous empirical research has found a positive correlation between the two dimensions. Perceived value is a direct predictor of program loyalty and is critical in establishing brand loyalty (Kim W. et al., 2018; Koo et al., 2020). Customers who perceive high value may become more dedicated to the company and attempt to recommend others to become loyal to the same company (Park and Baek, 2018). And the introduction of LPs is proven to increase customer retention and purchase intention by providing enhanced value (Shaikh et al., 2018). These arguments suggest that members’ perceived value is critical in forming different dimensions of loyalty. Thus, this study hypothesizes:

*H5:* Members’ perceived value has a positive influence on program loyalty.

*H6:* Members’ perceived value has a positive influence on store loyalty.

Previous research also suggests that each sub-dimension of perceived value impacts loyalty. The emotional connection between stores and customers is difficult to imitate by competitors, and the level of emotional value can significantly impact customer loyalty (Ahn and Thomas, 2020). Therefore, increasing customer emotional value is a very compelling market strategy in the customer-oriented new consumption era. High social value perception stimulates members to feel superior to non-members, can effectively improve their preference for LPs, and increases their willingness to maintain long-term contact with the stores (Koo et al., 2020). Various customer groups have placed a high value on the economic worth of LPs (Kang et al., 2015). They feel superior and believe the LPs will be more appealing when members consider their input is smaller than that of non-members, thus forming customer loyalty. According to Li and Lee (2016) and Chiu et al. (2014), there is a link between functional value and customer or brand loyalty. Based on the above discussions, we hypothesize the following:

*H5a-d:* Member perceptions of (a) functional value, (b) emotional value, (c) social value, and (d) economic value have a positive influence on program loyalty.

*H6a-d:* Member perceptions of (a) functional value, (b) emotional value, (c) social value, and (d) economic value have a positive influence on store loyalty.

Member perceived value is determined by their experience throughout the purchasing process, significantly impacting customer purchase intention and behavior. According to previous literature, perceived values and benefits accessible through LPs may cultivate loyalty toward the program, boosting loyalty to the store (Willems et al., 2017). Customers who perceive a higher degree of value are also more likely to achieve their shopping goals and show loyalty to the program, which can lead to attitudinal and behavioral loyalty to the retail store (Roy et al., 2017). In this paper, we predict that members’ loyalty perception toward the program mediates the effect of value perception on their formation of loyalty toward the store. We hypothesize as follows:

*H7:* Program loyalty mediates the relationship between perceived value and store loyalty.

*H7a-d:* Program loyalty mediates the relationship between (a) functional value, (b) emotional value, (c) social value, and (d) economic value and store loyalty.

### Definition and research hypotheses of alternative attractiveness

Previous literature defines alternative attractiveness as customers’ perceptions and assessments of viable competing alternatives, the heterogeneity among alternatives, and the benefits and costs of switching (Chuah et al., 2017; Singh and Rosengren, 2020). Alternative attractiveness significantly affects the consumer decision-making on whether to continue shopping at the store or using the LPs (Pick and Eisend, 2013). Customers will switch to those stores if competitors are considered more appealing, dependable, and offering more valuable goods and services (Chan et al., 2022). However, suppose customers perceive psychological or financial costs connected with leaving the existing store. In that case, they may be less likely to visit competitors and more likely to spend more there (Kim M. et al., 2018). According to cognitive dissonance theory, customers’ decision-making is easy and cognitive dissonance is unlikely to manifest itself when alternative options appear less desirable or attractive. However, that is not the case when the alternatives are similar (Tanford and Montgomery, 2014).

**Figure 1:**
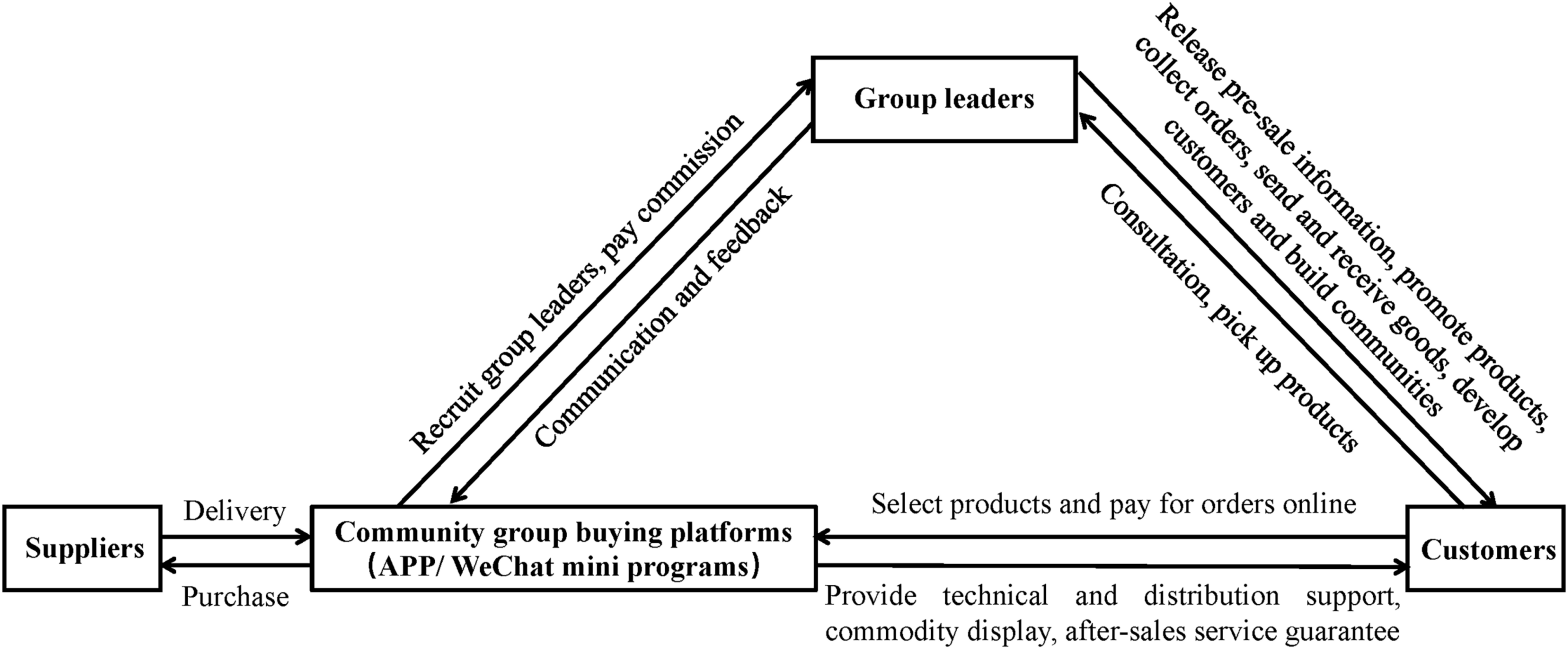
Operation process of community group buying.

With the common attributes of social e-commerce and social media, community group buying adopts the “pre-sale + collection and sale + last-mile delivery + self-pickup at the store” and sells everyday necessities and life services (Wang and Qiu, 2020; Li et al., 2022). Similar to the traditional convenience store operation concept, community group buying (Figure 1 shows its operation model) takes advantage of the acquaintance economy in the offline physical community (Liu et al., 2021; Ou et al., 2022). Therefore, they can achieve a higher-order rate and eliminates intermediate links to control costs. The advent of community group buying has further reduced convenience stores’ market share. As a result, many stores are increasing customer loyalty and obtaining new customers from competitors by implementing new social marketing strategies (such as virtual communities) or enhancing current marketing tools (such as LPs). Earlier research mainly looks into the influence of alternative attractiveness on the development of customer loyalty in conventional market settings (Chuah et al., 2017). The studies on the impact of community group buying in convenience store settings mainly focus on a qualitative analysis of the current situation (Wang and Qiu, 2020; Ou et al., 2022). In this paper, we select to study the attractiveness of community group buying as a critical determinant of the relationship between customer perceived value and loyalty formation. We define the attractiveness of community group buying as members’ perceptions and assessments regarding the relative advantages of community group buying and their willingness to buy products on community platforms instead of shopping at the store or using the LPs.

According to cognitive dissonance theory, appealing alternatives will lead to cognitive dissonance, further lowering perceived value and loyalty (Tanford and Montgomery, 2014). Numerous earlier studies prove that the alternative appeal moderates the connection between perceived value and customer loyalty (Chuah et al., 2017; Itani et al., 2019). In conclusion, we predict that the attractiveness of community group buying impacts the relationships between customers’ perceptions of the LPs’ value and loyalty. In other words, perceived value is more critical to customer loyalty when the attractiveness of community group buying is low; thus, we develop the following hypotheses.

*H8:* Alternative attractiveness negatively moderates the relationship between member perceived value and program loyalty.

*H8a-d:* Alternative attractiveness negatively moderates the relationship between (a) functional value, (b) emotional value, (c) social value, and (d) economic value and program loyalty.

*H9:* Alternative attractiveness negatively moderates the relationship between member perceived value and store loyalty.

*H9a-d:* Alternative attractiveness negatively moderates the relationship between (a) functional value, (b) emotional value, (c) social value, and (d) economic value and store loyalty.

### Model building

**Figure 2:**
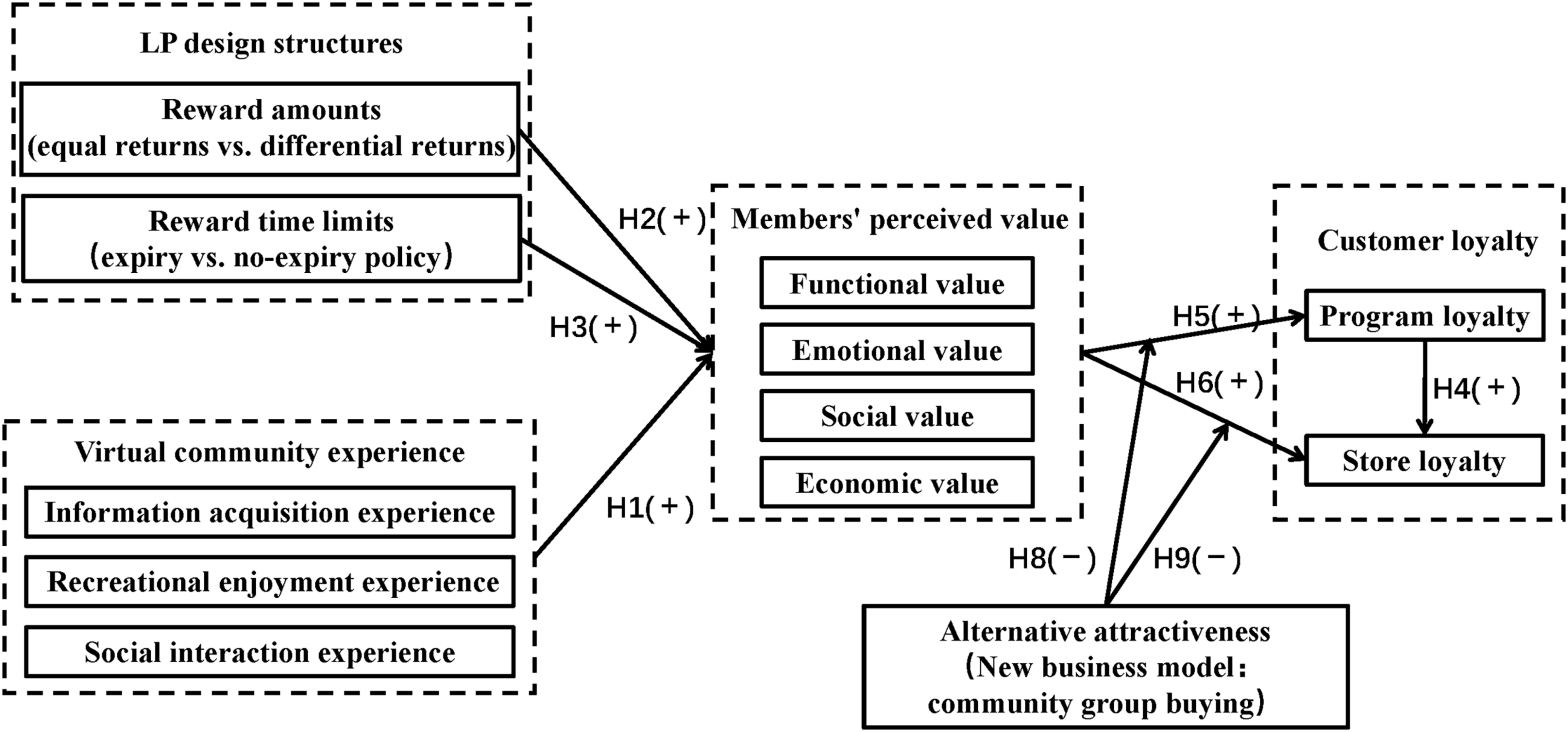
Conceptual model.

Based on the prior literature review and research hypotheses, this paper employed LP design structures and members’ virtual community experience as antecedent variables, members’ multidimensional perceived value as the intermediate variable, alternative attractiveness as the moderating variable, and customer loyalty as the outcome variable to investigate the influence path of “LP design structures + virtual community experience → perceived value → customer loyalty” relationship of traditional convenience stores (Figure 2 shows the conceptual model).

## Methodology and data collection

### Experimental design and measurements

This study adopted a 2 reward amounts (equal returns vs. differential returns) × 2 reward time limits (expiry vs. no-expiry policy) quasi-experimental design, and the reward amounts and reward time limits were manipulated (Appendix 1). Respondents were randomly assigned to one of the four experimental conditions. The questionnaire had three sections in addition to a brief description of the survey’s purpose. The first section was the screening of respondents. Only those who had ever been a member of the LPs at convenience stores, who had engaged in a virtual community and shopped at community group buying platforms, were permitted to continue with the survey. The second part was the main body of the questionnaire, including a detailed description of the specific LP scenarios and measurements of members’ virtual community experience, perceived value, alternative attractiveness, program loyalty, and store loyalty (Table 2). All items were measured on a 7-point Likert scale ranging from 1 = strongly disagree to 7 = strongly agree, using multiple-item scales adapted from previous literature, with only minor changes in the wording to suit the convenience store context adequately. The mean of the items was used to construct variables and test hypotheses. The last section included four socio-demographic questions (including demographic variables such as gender, age, education, and average monthly income, all of which were measured using categorical scales), two manipulation check questions, and two further survey questions (including “Do the convenience stores where you shop regularly engage in community group buying as self-pickup points?” and “Have you ever chosen convenience stores that regularly shopped as self-pickup points when shopping on community group buying platforms?”).

**Table 2:** Reliability and validity tests of variables.

Constructs and items	Factor loadings
**Information acquisition experience** from Hughes and Ahearne (2010) and Qiao et al. (2019) (α = 0.867, AVE = 0.568, CR = 0.868)	
IAE1: The shopping knowledge and product use experience acquired in the community greatly help my shopping.	0.710
IAE2: Comments or exchanges from other community members may help me solve many problems in purchasing products or participating in LPs.	0.738
IAE3: The information on LPs or products published in the community is rich, clear, and accurate.	0.754
IAE4: I can get the promotion information or member activities launched by the LPs in the community for the first time.	0.746
IAE5: Through the information released by this community, I am more familiar with this convenience store and its LPs and products.	0.815
**Recreational enjoyment experience** from Hughes and Ahearne (2010) and Huangfu et al. (2022) (α = 0.818, AVE = 0.604, CR = 0.821)	
REE1: I think the content (text, pictures, video, etc.) in this community is fascinating.	0.778
REE2: I think the membership activities organized by this community are rich and exciting.	0.735
REE3: I think the overall atmosphere of this community is comfortable, which can help me relieve pressure and forget worries.	0.817
**Social interaction experience** from Qiao et al. (2019) and Huangfu et al. (2022) (α = 0.880, AVE = 0.595, CR = 0.880)	
SIE1: Through this virtual community, I can make new friends with similar interests, expand my interpersonal circle and enrich my social life.	0.732
SIE2: I am willing to participate in the topic of community members.	0.803
SIE3: When I feel annoyed or bored, I want to spend time in the community.	0.760
SIE4: I am willing to share product knowledge, shopping experience, and LP use experience in the community and actively help community members.	0.769
SIE5: I can communicate with convenience stores at any time through this virtual community.	0.792
**Functional value** from Xie and Chen (2013) and Kang et al. (2015) (α = 0.847, AVE = 0.585, CR = 0.849)	
FV1: A variety of products can be purchased through this LP.	0.729
FV2: The quality of products purchased by the LP is reliable.	0.767
FV3: Continue to use the LP can get more value-added services.	0.740
FV4: Continuous use of this LP can lead to more reliable after-sales service.	0.820
**Emotional value** from Xie and Chen (2013) and Kang et al. (2015) (α = 0.852, AVE = 0.594, CR = 0.854)	
EV1: I feel happy and excited to participate in this LP.	0.786
EV2: I feel relaxed about using the LP to shop continuously.	0.698
EV3: I feel satisfied when I keep using the LP.	0.775
EV4: I enjoy using the LP to shop continuously.	0.818
**Social value** from Xie and Chen (2013) and Kang et al. (2015) (α = 0.830, AVE = 0.552, CR = 0.831)	
SV1: Continuing to shop with this LP makes me more popular.	0.727
SV2: Continuous use of this LP can increase my recognition.	0.738
SV3: Continuing to shop with this LP helps me make a good impression on others.	0.694
SV4: Continued participation in the LP makes me feel superior.	0.808
**Economical value** from Xie and Chen (2013) and Kang et al. (2015) (α = 0.854, AVE = 0.596, CR = 0.855)	
ECV1: I can get better prices through participating in this LP than other non-member customers.	0.756
ECV2: The LP gives me unique benefits and activities exclusive to members.	0.765
ECV3: I think shopping through this LP can reduce the time spent picking products, for it would recommend the most cost-effective products.	0.761
ECV4: I believe that continued use of the LP has a high cash value.	0.805
**Program loyalty** from Vilches-Montero et al. (2018) and Hwang et al. (2019) (α = 0.862, AVE = 0.616, CR = 0.865)	
PL1: I have a strong preference for this LP.	0.774
PL2: The LP can promote my consumption in the convenience store.	0.731
PL3: I would recommend this LP to my friends and relatives.	0.784
PL4: I have the intention to continue shopping with this LP.	0.846
**Store loyalty** from Gupta et al. (2018) and Hwang et al. (2019) (α = 0.862, AVE = 0.615, CR = 0.864)	
SL1:This convenience store is still my first choice when I have shopping needs in the future.	0.794
SL2:Although the price of some products in other stores is lower, I still prefer this convenience store.	0.720
SL3: I would recommend this convenience store to my friends and relatives.	0.787
SL4:I will encourage my friends and relatives to shop at this convenience store.	0.832
**Alternative attractiveness** from Picón et al. (2014) and Kim M. et al. (2018) (α = 0.764, AVE = 0.525, CR = 0.768)	
AA1: I think community group buying (e.g., Meituan Selected, TaoCaiCai, Xingsheng Selected, etc.) is also a good choice when I have shopping needs.	0.719
AA2: Compared with this convenience store, I think community group buying can also meet my shopping needs.	0.717
AA3: Compared with this convenience store, I am more satisfied with the products and services provided by community group buying.	0.737

α, Cronbach α; CR, composite reliability; AVE, average variance extracted values.

### Distribution and collection of questionnaires

Before conducting the final survey, two marketing research professionals, three experienced convenience store retailers, and five customers reviewed the draft questionnaire to ensure that the items were clear and relevant. After receiving the feedback, some minor changes were made to some items, removing ambiguity and improving understanding.

We conducted the final survey and recruited 559 participants on a professional platform named Credamo from November 2021 to December 2021, which can provide large-scale data collection services and has been recognized by top international journals. After eliminating 26 invalid surveys (All the answers to the measurement of items were 4 = uncertainty, and both answers for manipulation check questions were incorrect), we acquired 533 valid questionnaires. The effective rate was 95.35%, covering 171 prefecture-level cities in 31 provincial-level administrative regions in China, which can accurately and comprehensively describe the psychology and behavior of Chinese citizens. Table 3 shows the socio-demographic profiles of the respondents.

**Table 3:** The socio-demographic profiles of the respondents.

Demographic factors and categorical scale		Reward amounts				Reward time limits				In total	
		Equal returns		Differential returns		Expiry policy		No-expiry policy			
		*N*	%	*N*	%	*N*	%	*N*	%	*N*	%
Gender	Female	135	49.63	124	47.51	137	48.93	122	48.22	259	48.59
	Male	137	50.37	137	52.49	143	51.07	131	51.78	274	51.41
Age	[18, 30]	74	27.21	89	34.10	86	30.71	77	30.43	163	30.58
	[31, 40]	75	27.57	66	25.29	74	26.43	67	26.48	141	26.45
	[41, 50]	67	24.63	58	22.22	65	23.21	60	23.72	125	23.45
	[51, 70]	56	20.59	48	18.39	55	19.64	49	19.37	104	19.51
Education	1	46	16.91	54	20.69	47	16.79	53	20.95	100	18.76
	2	59	21.69	73	27.97	64	22.86	68	26.88	132	24.77
	3	108	39.71	99	37.93	122	43.57	85	33.60	207	38.84
	4	59	21.69	35	13.41	47	16.79	47	18.58	94	17.64
Average monthly income (CNY)	<3,000	64	23.53	56	21.46	57	20.36	63	24.90	120	22.51
	[3,001, 5,000]	67	24.63	64	24.52	63	22.50	68	26.88	131	24.58
	[5,001, 8,000]	75	27.57	59	22.61	75	26.79	59	23.32	134	25.14
	[8,001, 10,000]	30	11.03	43	16.48	48	17.14	25	9.88	73	13.70
	>10,000	36	13.24	39	14.94	37	13.21	38	15.02	75	14.07

Education: 1, high school education or below; 2, college degree; 3, bachelor’s degree; 4, master’s degree or above.

## Statistical analysis and results

### Manipulation checks

The questionnaire asked participants to recall the LP structures mentioned at the beginning to check that the manipulated factors produced the desired effects. Participants need to answer the two items: “Which type of reward amounts do you acquire in this LP” and “Is there a time limit for rewarding benefits gained through this LP.” The manipulation of reward amount (0 = differential return, 1 = equal return) and reward time limits (0 = with time limits, 1 = without time limits) was checked with a dichotomous scale. Logistic regression was performed with the recalled reward amount (reward time limit) as the dependent variable and the manipulated reward amount (reward time limit) as the independent variable. The results indicated that 90.5% of participants correctly recalled the reward amount type in the LP with equal return condition, and 88.6% of participants correctly recalled in the LP with differential return condition. The main effect of the manipulated reward amount on the recalled reward amount is statistically significant [Wald Chi-Squared (532, 1) = 227.741, *p* < 0.001]. The results indicate that 89.1% of participants correctly recalled the reward time limits in the condition with an expiry policy, and 91.6% of participants correctly recalled in the condition with a no-expiry policy. The main effect of the manipulated reward time type on the recalled reward time limits is statistically significant [Wald Chi-Squared (532, 1) = 231.386, *p* < 0.001]. Therefore, the manipulations of reward amounts and reward time limits were effective.

### Descriptive statistical analysis of variables

Table 4 shows the mean, standard deviation, and Pearson correlation matrix of the variables measured in this study. To assess the strength and direction of the relationships between variables and the multicollinearity of the independent variables, we adopted the Pearson correlation matrix and variance inflation factors (VIF; Hair et al., 2003). Although all variables were substantially correlated, the VIFs were all less than 10 (Tamhane and Dunlop, 2000), indicating that multicollinearity was not a serious concern.

**Table 4:** Mean, standard deviation, Pearson correlation matrix, and variance inflation factors of variables.

	M	SD	IAE	REE	SIE	SV	EV	FV	ECV	AA	PL	SL
IAE	5.829	0.810	1									
REE	5.728	0.925	0.441^**^	1								
SIE	5.844	0.884	0.509^**^	0.569^**^	1							
SV	5.797	0.858	0.481^**^	0.551^**^	0.586^**^	1						
EV	5.796	0.874	0.533^**^	0.578^**^	0.593^**^	0.615^**^	1					
FV	5.782	0.877	0.577^**^	0.570^**^	0.583^**^	0.572^**^	0.621^**^	1				
ECV	5.782	0.863	0.555^**^	0.573^**^	0.572^**^	0.583^**^	0.656^**^	0.662^**^	1			
AA	5.681	0.862	0.481^**^	0.487^**^	0.594^**^	0.565^**^	0.574^**^	0.526^**^	0.599^**^	1		
PL	5.777	0.888	0.526^**^	0.582^**^	0.614^**^	0.543^**^	0.648^**^	0.662^**^	0.683^**^	0.623^**^	1	
SL	5.836	0.908	0.531^**^	0.541^**^	0.616^**^	0.585^**^	0.672^**^	0.659^**^	0.671^**^	0.596^**^	0.708^**^	1
VIF			1.733	1.927	2.098	2.016	2.423	2.446	2.557	2.547	1.733	-
Square root of AVE			0.754	0.777	0.772	0.743	0.771	0.765	0.772	0.724	0.785	0.784

IAE, information acquisition experience; REE, recreational enjoyment experience; SIE, social interaction experience; SV, social value; EV, emotional value; FV, functional value; ECV, economic value; AA, alternative attractiveness; PL, program loyalty; SL, store loyalty; M, mean; SD, standard deviation; VIF, variance inflation factors.

** *p* < 0.01.

### Reliability and validity test for questionnaire

Given that all items in this paper were measured using established scales, this section employed AMOS 24.0 to conduct a confirmatory factor analysis (CFA) of the measurement model to examine the questionnaire’s reliability and validity and the model’s fitness. The standard values of several fitted indicators are within the acceptable range, indicating that the model is a good fit (see Model 1 in Table 5).

**Table 5:** Model fitting index of confirmatory factor analysis.

Index	χ^2^/df	GFI	IFI	TLI	NFI	CFI	RMSEA	AIC	BIC
Evaluation criterion	<3	>0.9	>0.9	>0.9	>0.9	>0.9	<0.05	As small as possible	
Model 1	1.474	0.922	0.974	0.970	0.923	0.974	0.030	1274.24	1809.06
Model 2	4.558	0.718	0.789	0.777	0.745	0.789	0.082	3533.22	3875.50
Model 3	2.229	0.894	0.935	0.923	0.888	0.934	0.048	1793.14	2443.48

Model 1, A multi-factor model without common method bias; Model 2, A one-factor model with common method bias only; Model 3, A multi-factor model with common method bias.

As shown in Table 2, the reliability coefficients (Cronbach α) of all variables (between [0.764, 0.880]) and the overall scale (0.966) were all greater than 0.70, indicating good reliability of the data (Nunnally and Bernstein, 1994). All measured items had standardized factor loadings between [0.764, 0.880], all above the threshold of 0.50 (Hair et al., 2010; Chai et al., 2015), showing good internal consistency of the scale. The composite reliability (CR) for all latent variables was between [0.768, 0.880], all exceeding the recommended criterion of 0.70; the average extracted variance values (AVE) for each latent variable were between [0.525, 0.616], all exceeding the threshold of 0.50, indicating strong convergent validity of the scale (Fornell and Larcker, 1981). The square root of AVE for each variable was bigger than its correlation coefficient with any other variable (Table 4), indicating that the scale also had good discriminant validity (Fornell and Larcker, 1981). The present scale offered good reliability and validity.

### Testing for common method bias

Common method bias was an artificial covariation between predictor and effector variables caused by the same data source (from the same questionnaire), a single survey population (all convenience store customers), and a single project research approach (focused on a single point in time rather than multiple longitudinal surveys; Podsakoff et al., 2003; Chai et al., 2015). It potentially threatened the research data’s validity and might mislead the study’s findings and conclusions. This research focused on procedural control methods and potential error variable control approaches to limit the influence of common method bias. To protect the anonymity of the respondents and limit the guesswork of the measuring purpose, this paper employed anonymous measurement and altered the order of the questions in the questionnaire to mix the questions describing different variables together in the procedural control. The potential error variable control method was to add common method bias as a latent variable to the model for confirmatory factor analysis. Thus we adopt a single-factor model with common method bias and a multi-factor model with common method bias. The fit of both the single-factor model (Model 2 in Table 5) and the multi-factor model (Model 3 in Table 5) became worse when compared to the results of the model without common method bias (Model 1 in Table 5), and the comparison of the main fit indices of Model 3 and Model 1 yields: △RMSEA = 0.02 < 0.05; △CFI = 0.04 < 0.1; △IFI = 0.03 < 0.1; and △TLI = 0.05 < 0.1 (Kline, 2011). In conclusion, this analysis found no significant common method bias.

### Comparative analysis of LPs with different design structures

The effectiveness of LPs with different reward amounts and reward time limits on members’ perceived value was investigated to test hypotheses H2a-d and H3a-d. We chose the Shapiro–Wilk test for normality analysis and the Levene test for Chi-square analysis of the data. According to the findings, the data did not follow a normal distribution (*p* < 0.05) but fulfilled the variance Chi-square criteria (*p* > 0.05); thus, the Mann–Whitney *U*-test was used. As shown in Table 6, LPs with differential returns had a more substantial effect on four dimensions of perceived value than equal returns. Thus hypotheses H2a-d were verified. Compared to the no-expiry policy, the effect of the expiry policy on four dimensions of perceived value was more substantial. Thus hypotheses H3a-d were confirmed.

**Table 6:** Non-parametric tests for the effectiveness of LPs with different reward amounts and reward time limits.

Constructs	Reward amounts	M	SD	U (×10^4^)	Z	Reward time limits	M	SD	U (×10^4^)	Z
FV	Equal	5.342	0.851	5.787^***^	12.651	Expiry	5.991	0.703	2.606^***^	−5.296
	Differential	6.241	0.636			No-expiry	5.551	0.988		
EV	Equal	5.354	0.833	5.803^***^	12.750	Expiry	5.987	0.725	2.709^***^	−4.722
	Differential	6.258	0.650			No-expiry	5.586	0.973		
SV	Equal	5.359	0.793	5.794^***^	12.695	Expiry	5.996	0.718	2.613^*^	−5.260
	Differential	6.255	0.662			No-expiry	5.578	0.944		
ECV	Equal	5.334	0.840	5.848^***^	13.004	Expiry	5.969	0.710	2.695^***^	−4.800
	Differential	6.250	0.600			No-expiry	5.576	0.966		

U, Mann–Whitney *U*-test statistics.

*** *p* < 0.001 and

* *p* < 0.05.

### Model evaluation and hypotheses testing

The main effect’s structural equation model was analyzed with AMOS 24.0 software, which contributed to understanding the impact of different dimensions of virtual community experience on perceived value in one model. To assess the mediation and moderation effects, we conducted a mediated moderation analysis following the bootstrapping method (with 5,000 iterations) with PROCESS 3.4 software, which could analyze a variety of mediation, moderation, and combination models (Hayes, 2018). Still, we could only put one independent variable in the model. Finding the appropriate process-set model for direct calculation was impossible due to the mode’s complexity. This paper analyzed the analysis of mediation and moderation effects based on process-set Models 4 and 8, with syntactic modifications and adjustments, and merged the virtual community experience into one variable (Appendix 2).

#### Structural equation modeling of main effects

The study conducted a structural equation model test of the main effects using Amos 24.0 to test the hypotheses. The fit indices of the model met the corresponding criteria (χ^2^/df = 1.921, GFI = 0.902, IFI = 0.952, TLI = 0.947, NFI = 0.905, CFI = 0.952, RMSEA = 0.042), indicating a good overall fit of the model. Table 7 showed that information acquisition experience significantly influenced four dimensions of perceived value (*p* < 0.001), and the hypothesis H1a-d remained true. Social interaction experience had a very significant positive influence (*p* < 0.001) on functional value, emotional value, and social value, and the hypotheses H1e-g were confirmed. Recreational enjoyment experience had a very significant favorable influence on emotional value (*p* < 0.001), supporting hypothesis H1h. Hypothesis H4 held because program loyalty significantly positively affected store loyalty (*p* < 0.001). Hypotheses H5a-b and H5d remained true because functional, emotional, and economic value significantly positively affected program loyalty (*p* < 0.001). However, the standardized path’s coefficient of social value on program loyalty is 0.012 (*p* > 0.05), indicating that hypothesis H5c was not supported. Emotional value had a highly significant positive effect on store loyalty (*p* < 0.001), supporting hypothesis H6b. Both functional and economic value significantly positively affected store loyalty (*p* < 0.01), and hypotheses H6a and H6d held. Social value had a relatively significant positive effect on store loyalty (*p* < 0.05), supporting hypothesis H6c.

**Table 7:** Test of the main effects and hypotheses.

Hypotheses	Path	Standardized coefficient	t	Hypotheses	Path	Standardized coefficient	t
H1a	IAE → FV	0.509^***^	9.456	H5a	FV → PL	0.319^***^	5.675
H1b	IAE → EV	0.341^***^	6.421	H5b	EV → PL	0.326^***^	6.010
H1c	IAE → SV	0.338^***^	6.056	H5c	SV → PL	0.012	0.234
H1d	IAE → ECV	0.748^***^	14.920	H5d	ECV → PL	0.333^***^	6.944
H1e	SIE → FV	0.385^***^	7.477	H6a	FV → SL	0.160^**^	2.828
H1f	SIE → EV	0.329^***^	5.483	H6b	EV → SL	0.251^***^	4.496
H1g	SIE → SV	0.501^***^	8.508	H6c	SV → SL	0.119^*^	2.499
H1h	REE → EV	0.255^***^	4.289	H6d	ECV → SL	0.161^**^	3.254
H4	PL → SL	0.326^***^	4.602				

*** *p* < 0.001;

** *p* < 0.01;

* *p* < 0.05.

#### Process-based mediation analysis

The analysis results (Table 8) of the mediation role of program loyalty proved that functional value, emotional value, and economic value all had a positive effect on program loyalty (*p* < 0.001) and store loyalty (*p* < 0.001). Social value had a non-significant effect on program loyalty (*p* > 0.05) but had a positive effect on store loyalty (*p* < 0.001). Program loyalty positively affected store loyalty (β = 0.303, *p* < 0.001). The mediation effect was tested by applying bootstrapping analysis (*N* = 5,000; Shrout and Bolger, 2002). As shown in Table 9, the confidence intervals for all three mediation paths did not contain zero, indicating that program loyalty partially mediated the relationship between emotional value, functional value, and economic value. Thus hypotheses H7a-b and H7d were supported. In contrast, hypothesis H7c was invalid because the confidence interval included zero. According to a comparison of the mediation effects of different paths as a proportion of the total effect, program loyalty had the most substantial mediation effect on the relationship between economic value and store loyalty with a proportion of 36.457%, and the weakest mediation effect on the relationship between emotional value and store loyalty with a proportion of 25.789%.

**Table 8:** Results of mediation analysis.

Independent variable	Model 4 (Outcome: PL)			Model 5 (Outcome: SL)		
	β	SE	t	β	SE	t
FV	0.279^***^	0.041	6.765	0.173^***^	0.041	4.169
EV	0.242^***^	0.042	5.718	0.211^***^	0.042	5.035
SV	0.059	0.040	1.486	0.116^***^	0.038	3.043
ECV	0.320^***^	0.044	7.371	0.169^***^	0.044	3.854
PL				0.303^***^	0.042	7.241
R^2^	0.580			0.629		
F	182.570^***^			178.650^***^		

*** *p* < 0.001.

**Table 9:** Bootstrapping analysis results of the mediation effect.

Path and hypotheses	Effect size	Boot SE	Boot 95%CI		Proportion of the total effect (%)
			Low	High	
H7a: FV → PL → SL	0.025	0.006	0.014	0.038	32.825
H7b: EV → PL → SL	0.019	0.006	0.009	0.031	25.789
H7c: SV → PL → SL	0.004	0.003	−0.002	0.012	13.353
H7d: ECV → PL → SL	0.023	0.006	0.012	0.035	36.457

#### Process-based moderation analysis

The findings (Table 10) revealed that alternative attractiveness had a negative moderation effect on the relationship between functional value and program loyalty(β = −0.023, *p* < 0.001) and on the relationship between functional value and store loyalty(β = −0.017, *p* < 0.01). The index of moderated mediation was significant (β_FV → PL → SL_ = −0.002, BootSE = 0.001, Boot 95%CI = [−0.004, −0.001]). Alternative attractiveness had a negative moderation effect on the relationship between emotional value and program loyalty (β = −0.027, *p* < 0.001) and on the relationship between emotional value and store loyalty (β = −0.016, *p* < 0.05). The index of moderated mediation was significant (β_EV → PL → SL_ = −0.003, BootSE = 0.001, Boot 95%CI = [−0.005, −0.002]). Alternative attractiveness had a negative moderation effect on the relationship between economic value and program loyalty (β = −0.026, *p* < 0.001) and on the relationship between economic value and store loyalty (β = −0.014, *p* < 0.05). The index of moderated mediation was significant (β_ECV → PL → SL_ = −0.003, BootSE = 0.001, Boot 95%CI = [−0.004, −0.001]). Alternative attractiveness had a negative moderation effect on the relationship between social value and store loyalty (β = −0.014, *p* < 0.05) but did not have a significant influence on the relationship between social value and program loyalty (*p* > 0.05). The index of moderated mediation was not significant (β_SV → PL → SL_ = −0.002, BootSE = 0.001, Boot 95%CI = [−0.002, 0.001]). Hence, hypotheses H8a-b,d, and H9a-d were supported, but hypothesis H8c was not.

**Table 10:** Moderation effect of alternative attractiveness.

Independent variable	Model 6 (Outcome: PL)			Model 7 (Outcome: SL)		
	β	SE	t	β	SE	t
FV	0.243^***^	0.041	5.944	0.160^***^	0.042	3.824
EV	0.190^***^	0.042	4.541	0.193^***^	0.042	4.559
SV	0.002	0.041	0.050	0.092^*^	0.041	2.283
ECV	0.246^***^	0.045	5.526	0.148^***^	0.045	3.265
PL				0.268^***^	0.043	6.188
AA	0.304^***^	0.051	5.974	0.136^***^	0.052	2.602
FV × AA	−0.023^***^	0.006	−3.629	−0.017^**^	0.006	−2.762
EV × AA	−0.027^***^	0.007	−4.140	−0.016^*^	0.006	−2.565
SV × AA	−0.003	0.015	−0.214	−0.014^*^	0.007	−2.048
ECV × AA	−0.026^***^	0.007	−3.927	−0.014^*^	0.007	−2.196
R^2^	0.612			0.638		
F	91.625^***^			91.938^***^		

*** *p* < 0.001;

** *p* < 0.01;

* *p* < 0.05.

A simple slope test was also used to uncover alternative attractiveness’s moderation effect (Table 11). Although the effects of functional value, emotional value, and economic value on program loyalty or store loyalty were positive in both the high and low alternative attractiveness conditions, the effects were more significant in the low condition compared to the high condition, supporting hypotheses H8a-b, H8d, H9a-b, and H9d. Although the effect of social value on store loyalty was positive in both the high and low alternative attractiveness conditions, the effect was more significant in the low condition compared to the high condition, further supporting hypothesis H9c. The simple slope test using PROCESS software did not report non-significant results, indicating that hypothesis H8c was not supported.

**Table 11:** The simple slope test for the moderation effect.

Moderating path	Categories	Effect size	SE	*t*	Moderating path	Effect size	SE	*t*
FV → PL	Low AA	0.484^***^	0.035	13.886	FV → SL	0.329^***^	0.040	8.275
	High AA	0.366^***^	0.045	8.178		0.240^***^	0.046	5.178
EV → PL	Low AA	0.473^***^	0.038	12.503	EV → SL	0.359^***^	0.041	8.836
	High AA	0.332^***^	0.045	7.323		0.275^***^	0.045	6.129
ECV → PL	Low AA	0.518^***^	0.038	13.719	ECV → SL	0.331^***^	0.043	7.676
	High AA	0.385^***^	0.047	8.158		0.257^***^	0.049	5.233
SV → PL	Low AA	-	-	-	SV → SL	0.266^***^	0.040	6.743
	High AA	-	-	-		0.194^***^	0.045	4.305

SE, standard error; Low AA, the low value of alternative attractiveness; High AA, the high value of alternative attractiveness. We did not report the data for the relationship between social value and program loyalty because the simple slope test using PROCESS software did not report the non-significant results. ***p < 0.001.

We further analyzed the moderation effect of alternative attractiveness under different conditions. As shown in Table 12, the moderation effect of alternative attractiveness was significant in the LP conditions with equal returns and no-expiry policy but not in the LP conditions with differential returns and expiry policy. It further evidenced that LPs with equal returns are more susceptible to the attractiveness of alternatives when compared to that with differential returns; and that LPs with a no-expiry policy are more susceptible to the attractiveness of alternatives when compared to that with differential returns.

**Table 12:** Moderation effect of alternative attractiveness under different conditions.

Different conditions	Independent variable	Model 8 (Outcome: PL)			Model 9 (Outcome: SL)		
		β	SE	t	β	SE	t
Equal returns	FV × AA	−0.030^**^	0.010	−3.171	−0.026^**^	0.010	−2.667
	EV × AA	−0.034^***^	0.010	−3.551	−0.026^**^	0.010	−2.715
	SV × AA	−0.003	0.021	−0.128	−0.024^*^	0.010	−2.353
	ECV × AA	−0.041^***^	0.010	−4.184	−0.026^**^	0.010	−2.606
Differential returns	FV × AA	−0.002	0.025	−0.066	−0.056^*^	0.026	−2.164
	EV × AA	−0.002	0.027	−0.074	−0.015	0.027	−0.543
	SV × AA	−0.010	0.026	−0.373	−0.021	0.027	−0.790
	ECV × AA	0.052	0.029	1.773	0.046	0.030	1.534
Expiry policy	FV × AA	−0.025	0.063	−0.387	−0.068	0.056	−1.218
	EV × AA	−0.068	0.061	−1.107	−0.014	0.052	−0.270
	SV × AA	−0.156^*^	0.068	−2.285	−0.009	0.057	−0.152
	ECV × AA	−0.074	0.058	−1.277	−0.020	0.052	−0.380
No-expiry policy	FV × AA	−0.097^***^	0.027	−3.630	−0.059^***^	0.028	−2.106
	EV × AA	−0.095^***^	0.026	−3.625	−0.064^*^	0.027	−2.351
	SV × AA	−0.106^***^	0.031	−3.414	−0.054	0.031	−1.782
	ECV × AA	−0.107^***^	0.027	−3.936	−0.062^*^	0.029	−2.114

This table only showed the results of the interaction effect.

*** *p* < 0.001;

** *p* < 0.01;

* *p* < 0.05.

### Effectiveness of the “convenience store + community group buying” model

The survey also investigated whether convenience stores where respondents frequently shopped participated in community group buying as self-pickup points and whether respondents had chosen these stores as self-pickup points when shopping on community group buying platforms. Its purpose was to see if these factors impacted perceived value, the formation of customer loyalty, and the perception of alternative attractiveness. All the participants had shopping experiences in the convenience store and community group buying platforms. In the investigation on whether convenience stores that shopped regularly participated in community group buying, 255 respondents gave an affirmative answer, and 278 gave a negative answer. Among the 255 respondents who reacted yes, 137 respondents said they had ever used them as self-pickup points, while 118 said no. We then adopted the Shapiro–Wilk test to analyze the data for normality and the Levene test for chi-square analysis. The results showed that the data did not follow a normal distribution (*p* < 0.05), but it did meet the Chi-square criteria (*p* > 0.05), then the Mann–Whitney *U-*test was used (Table 13). Members’ emotional value, economic value, program loyalty, and store loyalty to the participating convenience store were all significantly enhanced. At the same time, their perception of the alternative role of community group buying was reduced compared to stores that did not participate. Customers who had chosen the stores as self-pickup points had significantly increased their perception of perceived value and loyalty and significantly reduced their perception of the alternative role of community group buying compared to customers who had not chosen.

**Table 13:** Effects of whether convenience stores participated in community group buying and whether selecting them as self-pickup points on different variables.

Constructs	Categories	M	SD	U (×10^4^)	Z	Categories	M	SD	U (×10^3^)	Z
SV	1	5.862	0.790	3.343	−1.142	3	6.062	0.726	5.575^***^	−4.297
	2	5.738	0.913			4	5.629	0.801		
EV	1	5.884	0.871	3.122^*^	−2.393	3	6.075	0.721	6.100^**^	−3.399
	2	5.716	0.871			4	5.663	0.975		
FV	1	5.833	0.853	3.295	−1.414	3	6.020	0.686	6.100^**^	−3.393
	2	5.736	0.898			4	5.617	0.971		
ECV	1	5.914	0.852	2.867^***^	−3.839	3	6.053	0.665	6.751^*^	−2.285
	2	5.662	0.857			4	5.752	1.007		
PL	1	5.849	0.902	3.171^*^	−2.117	3	6.088	0.714	5.669^***^	−4.136
	2	5.710	0.872			4	5.572	1.014		
SL	1	5.951	0.890	3.124^**^	−3.060	3	6.183	0.709	5.662^***^	−4.149
	2	5.731	0.914			4	5.682	1.000		
AA	1	5.780	0.827	3.124^*^	2.388	3	5.978	0.760	5.751^***^	4.008
	2	5.590	0.884			4	5.551	0.845		

Categories 1, Convenience stores participated in community group buying; 2, Convenience stores did not participate; 3, Respondents had used the frequent shopping convenience stores as self-pickup points; 4, Respondents had not used. ***p < 0.001; **p < 0.01; *p < 0.05.

## Conclusion and discussion

### Key findings

Chosen traditional convenience stores as the research object, this paper takes different LP design structures (reward amounts × reward time limits) and the three-dimensional virtual community experience as the antecedent factors, the four-dimensional perceived value as the intermediate variable, and the two-dimensional customer loyalty as the outcome variable. This paper compares the effectiveness of different LP design structures using a non-parametric test. It also investigates the effect of members’ virtual community experience on perceived value and customer loyalty using AMOS and PROCESS software. In summary, we identify five critical findings as follows.

First, we discover that LPs with different structures significantly differ in customer value perception. LPs with differential returns are more effective in increasing members’ perception of value than those with equal returns, consistent with Steinhoff and Palmatier (2016), due to the pressure to upgrade or retain a current membership level to get more valuable rewards. LPs with an expiry policy are more effective than the no-expiry policy, which are consistent with those of Dorotic et al. (2014) and Bazargan et al. (2017), owing to the time pressure mechanism created by the reward time limit. According to the target gradient theory, as the next level’s threshold or the expiration date approaches, the level shift and the time limit produce a psychological pressure mechanism for members, increasing their motivation to continue participating in LPs and increasing purchases.

Second, a good experience in the virtual community is confirmed to be beneficial to customers’ perception of the benefits and value of the LPs. The information acquisition experience has a considerable positive influence on the four dimensions of perceived value. However, the degree of influence varies with the most substantial influence on economic value and the slightest influence on social and emotional value. The social interaction experience has a significant favorable impact on three dimensions: functional, emotional, and social value, but the degree of impact varies, with social value having the most significant impact. The recreational enjoyment experience has a substantial favorable impact on emotional value. The findings are consistent with those of Kim et al. (2018a) and Li et al. (2022), possibly because the perceived value of LPs is customers’ subjective feeling after weighing the costs and benefits, and the virtual community experience is also a psychological feeling in the process of participating in the community. The information customers receive in the community, such as detailed product descriptions and promotion notices, primarily assists them in fully understanding the promotion rules and the products they need before purchasing. It also allows them to make the best decision possible, which dramatically reduces the purchase cost and time cost and thus has the most significant impact on increasing their economic value. Customers can communicate with retailers at any time and from anywhere *via* the community and form connections with other members, dramatically expanding their social circle and increasing their sense of belonging, thus having the most significant impact on enhancing their social value. Retailers’ sharing of product-related suggestions and store anecdotes in the community and emotional communication with other members can strengthen customers’ senses, emotions, and cognition, thus boosting their perception of emotional value.

Third, we find the critical role of perceived value in the formation mechanism of customer loyalty. Functional, emotional, and economic values have significantly influenced program and store loyalty, with emotional value having the most significant impact on store loyalty. The findings are consistent with the work of Ahn and Thomas (2020), suggesting that member loyalty is influenced not only by the perceptions of the product price and quality, various promotions, and exclusive member services through participation in LPs but, more importantly, the emotional satisfaction and happiness received. Inconsistent with our hypotheses, social value does not affect program loyalty and only has a weak effect on store loyalty, consistent with the studies of Yang and Mattila (2016). Customers with a high perception of social value are most likely to leave the LP and go private, for LPs in convenience stores do not facilitate the flow of resources among customers and have limited ability to improve their status perceptions. Thus the perception of social value in the convenience store environment does not drive customers’ continued participation in the program and increase purchases in the store.

Fourth, program loyalty has a direct effect on store loyalty and a mediating effect on the relationship between perceived value and store loyalty. The results reveal that, in addition to social value, program loyalty partially mediates the relationship between functional, emotional, and economic value with store loyalty. The finding is in line with the findings of Roy et al. (2017), which state that retailers need to implement LPs and build exclusive member service delivery platforms, continuously providing members with high-value benefits and building strong emotional ties. Program loyalty also has the most substantial mediation effect on the link between economic value and store loyalty. In contrast, it has the weakest mediation effect on the relationship between emotional value and store loyalty. In summary, we conclude that the economic benefits perceived by members participating in a convenience store’s LPs have the most substantial contribution to store loyalty formation.

Finally, we discover the adverse moderating effects of the attractiveness of community group buying on the relationship between customers’ perceived value and loyalty, but the degree of impact is weak. Unlike the findings of previous studies, which advocate the strong influence of alternative attractiveness (Kim M. et al., 2018; Huangfu et al., 2022), we prove that the impact of community group buying on customer value drive and loyalty formation in the convenience stores scenario is limited. The first reason is that they focus on different types of products. The primary product of community group buying is fresh food, which most traditional convenience stores do not offer because of its low gross margin, high gloss, and lengthy supply chain. The second reason is that changes and advances in convenience stores can weaken the attractiveness of competitors. By introducing new social media marketing methods and designing more valuable LPs, convenience stores continuously improve customer service levels and optimize the products sold, thus enhancing customer loyalty and maintaining advantages in the market competition. A further survey also confirmed that a convenience store’s participation in community group buying and customers’ decision to use it as the self-pickup points increase customers’ perceived value and loyalty while lowering their perceptions of alternative attractiveness.

### Theoretical contributions

This study contributes to the literature in four ways. First, our research adds to LP and loyalty literature by exploring the effectiveness of LPs with different structures in enhancing members’ value and loyalty. Recent studies have emphasized the effectiveness of linear LPs in customer loyalty formation rather than nonlinear (multi-tier) LPs (Yang et al., 2019, 2021) and paid little attention to the effect of reward duration. This paper compared LPs with different reward amounts (equal returns vs. differential returns) and reward time limits (expiry vs. no-expiry policy) for their impact on perceived value. The results provide new insights and theoretical support for how convenience stores should design LPs and play the role of LPs to retain customers.

Second, our study is one of the very few empirical tests that verify the effects of three dimensions of virtual community experience on four dimensions of perceived value in convenience store settings. The extant literature has intensively examined the influence of community experience on customer usage intention in the e-commerce context ( Foroudi et al., 2016; Lemon and Verhoef, 2016). However, little attention has been given to the multidimensional experience and value and the advantages of creating virtual communities in the context of convenience stores. Our findings highlight the importance of improving members’ virtual community experiences in promoting customer perceived value and show that each type of community experience impacts customer perceived value differently. Thus, our research enriches the existing literature research system of perceived value and social media marketing literature and practice.

Third, we advance loyalty theory by exploring the internal linkage mechanisms of the relationship of perceived value-program loyalty-store loyalty. Unlike previous studies (e.g., Berezan et al., 2017; Bruneau et al., 2018), we unfold a single dimension of loyalty into two separate dimensions: program loyalty and store loyalty, showing that customer perceived value can, directly and indirectly, affect both types of loyalty with different magnitudes. We also show new insight into how different values can influence members’ loyalty toward the LPs and store and prove that emotional value significantly impacts store loyalty, but social value does not.

Finally, this research fills the gap of quantitative research on the moderating role of community group buying from the perspective of the convenience store. With the rapid development of community group buying, its impact on the real-community commercial and its attractiveness in consumer decision-making should be taken seriously (Li et al., 2022). We discover that community group buying does harm the relationship between consumer perceived value and loyalty, but the impact is negligible. The attraction may not be significant if the convenience store offers a high-value LP or introduces practical social media marketing tools. The findings enrich our understanding of this new business model with the attributes of community business and social media and help retailers better to play the role of social media marketing strategies.

### Practical implications

Our research offers several practical insights for convenience store retailers to motivate and engage their LP members more effectively. First, we recommend convenience stores empower traditional marketing techniques (LPs-based relationship marketing) with new social media marketing tools(virtual community-based marketing) to increase member activity and strengthen stickiness. This paper finds that different designed LPs have entirely different effects on customer value and loyalty cultivation. Thus convenience stores should pay attention to the structure and effectiveness of LPs. We also recommend Growth-oriented and Periodical zeroing LPs for retailers, which divide members into different levels based on their spending habits and encourage customers to reward the benefits as quickly as feasible. This paper also proves that creating a virtual community enhances customer value and loyalty. With the community’s help, retailers can build a communication bridge for members in the surrounding real communities and a private traffic pool for themselves. They can better advertise and promote LPs without increasing operating expenses, increase member involvement in LPs, and reactivate dormant members for secondary consumption. For example, retailers can break through time and space barriers to stay in touch with their members by creating a member-only virtual community. They can deliver product promotion information, share product usage advice, and recommend cost-effective products to members more quickly and effectively. Members can offer help, share shopping experiences, and exchange exciting life stories and practical life tips with other members. Members can also make friends with like-minded people and broaden their social circle to increase participation, enthusiasm, and loyalty toward the store.

Second, we suggest convenience stores pay more attention to the emergence of new competitors and build a “convenience store + community group buying” model. This study discovers that the emergence of community group buying harms the formation of member loyalty, but the effect is minor. It also discovers that convenience stores’ participation in community group buying improves perceptions of customer value and loyalty but reduces the perception of alternative attractiveness. Therefore, A “convenience store + community group buying” model is suggested to innovate and overcome the development challenge. (1) Convenience stores can participate in community group buying and become group leaders. They can help send and receive orders and share information about fresh products in their exclusive member community, which provides members with benefits and convenience and effectively compensates for the lack of fresh products. (2) Convenience stores can learn from the community group buying’s development advantages and concept features to offer exclusive services to members in their virtual communities. For example, they can utilize a pre-sale mode to collect orders, provide home delivery services, and conduct promotional activities such as group buying and price-cutting in the virtual community. These strategies may reduce operational and inventory expenses, expand sales categories, and boost members’ desire to buy and join. (3) Convenience stores can take advantage of the preferential policies of community group buying platforms to provide members with additional preferential rights. They can also use self-pickup to attract customers to their offline store, allowing them to experience their service and product quality and potentially become their customers or join their LPs.

### Limitations and future research

Although the findings of this paper have significant implications for both loyalty research and convenience store development, several limitations of our research warrant future research. First, we tested the causal relationships between the variables using cross-sectional data. Future research may conduct longitudinal investigations to see the changes in the relationship between members’ virtual community experience, perceived value, and loyalty. Second, this study only looks at the convenience store portion of the retail business and only collects data from a single market, China. Future studies should include data from different sectors or countries and make comparisons of the findings between different industries or nations to improve the generalizability of the suggested model and outcomes.

## Data availability statement

The raw data supporting the conclusions of this article will be made available by the authors, without undue reservation.

## Ethics statement

Ethical review and approval was not required for the study on human participants in accordance with the local legislation and institutional requirements. Written informed consent from the patients/participants or patients/participants legal guardian/next of kin was not required to participate in this study in accordance with the national legislation and the institutional requirements.

## Author contributions

XX organized the database, performed the statistical analysis, and wrote the first draft of the manuscript. ZH contributed to conception and design of the study. All authors contributed to the article and approved the submitted version.

## Conflict of interest

The authors declare that the research was conducted in the absence of any commercial or financial relationships that could be construed as a potential conflict of interest.

## Publisher’s note

All claims expressed in this article are solely those of the authors and do not necessarily represent those of their affiliated organizations, or those of the publisher, the editors and the reviewers. Any product that may be evaluated in this article, or claim that may be made by its manufacturer, is not guaranteed or endorsed by the publisher.

## Appendix 1: Questionnaire Scenario Description

1. Reward amounts-equal returns:
  There is no membership level and all members enjoy the same membership rights. Members can earn 2 points for every 1 yuan spent, which can be redeemed for gifts in the store’s online virtual community.
2. Reward amounts-differential returns:
  Membership level is set up, and membership benefits will change with the change of membership level. Regular members can get 1 point for every 1 CNY spent, silver card members can get 2 points for every 1 CNY spent, and gold card members can get 3 points for every 1 CNY spent. The points earned can be redeemed for gifts in the store’s online virtual community.
3. Reward time limits-expiry policy:
  Points accumulated must be redeemed by the end of the following year.
4. Reward time limits-no-expiry policy:
  There is no expiry date for points accumulated on purchases.

## Appendix 2: The syntax for mediation analysis and moderation analysis

1. The syntax for mediation analysis based on Process
  PROCESS
  y = SL /x = VCE /m = SV EV ECV FV PL /contrast = 1 /total = 1 /plot = 1 /covcoeff = 1 /decimals = F10.4 /moments = 1 /modelbt = 1 /effsize = 1 /stand = 1 /boot = 5,000 /xmtest = 1 /conf = 95 /bmatrix = 1,1,0,1,0,0,1,0,0,0,0,1,1,1,1,0,1,1,1,1,1.
2. The syntax for moderation analysis based on Process
  PROCESS
  y = SL /x = VCE /m = SV EV ECV FV PL /w = AA /contrast = 1 /total = 1 /plot = 1 /covcoeff = 1 /decimals = F10.4 /center = 1 /moments = 1 /modelbt = 1 /effsize = 1 /stand = 1 /boot = 5,000 /xmtest = 1 /conf = 95 /bmatrix = 1,1,0,1,0,0,1,0,0,0,0,1,1,1,1,0,1,1,1,1,1 /wmatrix = 0,0,0,0,0,0,0,0,0,0,0,1,1,1,1,0,1,1,1,1,0.
